# Resignation of Professor Warren

**Published:** 1847-05

**Authors:** 


### RECORD OF MEDICAL SCIENCE.

*Resignation of Professor Warren.*—It will be perceived from the following record, that Dr. John C. Warren has resigned the chair of anatomy and physiology, in Harvard University, Boston, which he has long filled with distinguished honour to himself, and great advantage to the school, and that the corporation of the University have shown their just appreciation of his valuable services by electing him Emeritus Professor of Anatomy and Surgery. In his retirement, the Professor will carry with him the best wishes of the profession, of which he has been a distinguished ornament, and for the advancement and improvement of which, we are sure, he will not cease to occupy himself. He has rich stores of experience to lay open, he is also engaged, we know, in scientific pursuits of great interest and importance, and we trust his life may be long spared for their completion, and to enjoy the honours he has justly earned.

At a stated meeting of the President and Fellows of Harvard University, in Boston, Feb 27th, 1847, the President laid before the corporation the following communication from Dr. Warren, resigning his Professorship.

[Here follows in the records a copy of Dr. Warren's letter.]

Whereupon voted—That in accepting the resignation of Dr. J. C. Warren, as Hersey Professor of Anatomy and Surgery, this board is deeply sensible of the important services rendered to the University by Dr. Warren; and holds in grateful recollection the successful exertions made by him, for a period of more than forty years, and in continuance of those of his honoured father, to raise the character, and promote the interests of the medical school.

Voted—That Dr. Warren be requested to continue in the discharge of the duties of his office, till the close of the present Academic year.

Voted—That the President be requested to communicate to Dr. Warren a copy of the foregoing votes, with the assurance that this board cordially reciprocates the friendly and respectful sentiments expressed towards the corporation and the University, in his letter of resignation.

On vote, by ballot, Dr. John C. Warren was chosen Emeritus Professor of Anatomy and Surgery in the University, in consideration of his faithful and valuable services as Hersey Professor of Anatomy and Surgery.

A true copy of the record.

Attest, JAMES WALKER, Secretary.

*Amer. Journ. Med. Sciences.*

